# SEXUAL ATTITUDES IN MIDDLE-AGED AND OLDER ADULTS: DEVELOPING AN UPDATED AND AGE-RELATIVE MEASUREMENT SCALE

**DOI:** 10.1093/geroni/igz038.1110

**Published:** 2019-11-08

**Authors:** Allyson S Graf, Viviane Johnson

**Affiliations:** Northern Kentucky University, Highland Heights, Kentucky, United States

## Abstract

Sexual attitudes in later life contribute to sexual and psychological well-being. Existing scales of sexual attitudes specific to aging adults are narrowly focused on specific settings or population sub-groups and are not inclusive of the diverse range of sex-related activities and sexual identities represented in present and upcoming older cohorts. The aim of this study was to create and validate a multi-dimensional measure of sexual attitudes appropriate for a sexually-diverse aging population. Following focus groups and feedback, 51 survey items were constructed; other measures of sexual attitudes, knowledge, and behaviors were included for the purposes of validation. A sample of participants (N = 291; Mage = 56.69, SD = 8.82, range = 45-99; 63.2% female) was recruited through Amazon’s Mechanical Turk and the local community to complete a survey on sexual attitudes, well-being, and health. Psychometric properties of the measure were assessed using exploratory factor analysis to reduce items and identify existing factor structures; construct validity was confirmed using correlations with existing measures of sexual attitudes; and internal consistency was assessed for each sub-scale. Five factors emerged: core personal values (8-items; α = .99), communication and expression (13-items; α = .92), sources of knowledge (9-items; α = .87), traditional taboos (9-items; α = .89), and consent within established relationships (4-items; α = .94). Results provide a relevant and multi-faceted measurement of sexual attitudes for use with middle-aged and older adult populations. Future studies may benefit from an alternative to dated, restrictive measures. Validation should be ongoing with more diverse samples.

